# Immunohistochemical Profiling of Histone Modification Biomarkers Identifies Subtype-Specific Epigenetic Signatures and Potential Drug Targets in Breast Cancer

**DOI:** 10.3390/ijms26020770

**Published:** 2025-01-17

**Authors:** Zirong Huo, Sitong Zhang, Guodong Su, Yu Cai, Rui Chen, Mengju Jiang, Dongyan Yang, Shengchao Zhang, Yuyan Xiong, Xi Zhang

**Affiliations:** 1 School of Life Science, Northwest University, Xi’an 710069, China; hzr1661864339@163.com (Z.H.); zstongoo@163.com (S.Z.); suguodong9123456@163.com (G.S.); caiyu1316@163.com (Y.C.); yourangle@126.com (R.C.); mengjur@outlook.com (M.J.); 202322544@stumail.nwu.edu.cn (D.Y.); zhangshengchao0601@163.com (S.Z.); yuyan.xiong@nwu.edu.cn (Y.X.); 2 School of Professional Studies, Northwestern University, Evanston, IL 60201, USA

**Keywords:** histone modification, immunohistochemical profiling, triple-negative breast cancer, G9a inhibitor

## Abstract

Breast cancer (BC) subtypes exhibit distinct epigenetic landscapes, with triple-negative breast cancer (TNBC) lacking effective targeted therapies. This study investigates histone biomarkers and therapeutic vulnerabilities across BC subtypes. The immunohistochemical profiling of >20 histone biomarkers, including histone modifications, modifiers, and oncohistone mutations, was conducted on a discovery cohort and a validation cohort of BC tissues, healthy controls, and cell line models. Transcriptomic and cell growth analyses were conducted to evaluate the effects of the small-molecule G9a inhibitor in diverse BC models. Key histone biomarkers, including H3K9me2, H3K36me2, and H3K79me, were differentially expressed across BC subtypes. H3K9me2 emerged as an independent predictor for distinguishing TNBC from other less-aggressive BC subtypes, with elevated expression correlating with higher tumor grade and stage. G9a inhibition impaired cell proliferation and modulated epithelial–mesenchymal transition pathways, with the strongest impact in basal-like TNBC. The disruption of the oncogene and tumor suppressor regulation (e.g., TP53, SATB1) was observed in TNBC. This study highlights G9a’s context-dependent roles in BC, supporting its potential as a therapeutic target. The findings provide a foundation for subtype-specific epigenetic therapies to improve outcomes in aggressive BC subtypes.

## 1. Introduction

Histones, as the core components of chromatin, undergo chemical modifications that regulate gene expression and are intimately linked to cancer development [1]. Histone modifications such as methylation and acetylation act as pivotal regulators of gene activity, influencing transcriptional regulation and tumor progression depending on their type and genomic context [2,3]. For instance, H3K4me2 is a mark of active transcription, typically found at promoters of genes primed for expression. Conversely, H3K9me2 is associated with transcriptional repression and heterochromatin formation, silencing genes that may drive tumorigenesis. However, the loss of H3K9me2 can paradoxically activate oncogenes, contributing to cancer progression.

BC is a highly heterogeneous disease, classified into distinct molecular subtypes based on the expression of classic biomarkers such as estrogen receptors (ERs), progesterone receptors (PRs), and Human Epidermal Growth Factor Receptor 2 (HER2) [4]. Among these, TNBC poses the greatest clinical challenge due to its aggressive nature and the absence of well-defined therapeutic targets. Bridging the treatment gaps in TNBC necessitates innovative approaches, particularly those focusing on its unique molecular and epigenetic characteristics [5]. Aberrations in histone modifications in cancer can vary significantly, even within the same cancer type. Research has demonstrated that different molecular subtypes of BC (e.g., luminal A and TNBC) exhibit distinct patterns of histone modifications, reflecting subtype-specific epigenetic landscapes [6]. Although evidence from cell line studies has highlighted that TNBC is the molecular subtype that is most closely associated with distinct histone modification patterns [7,8], research focusing on human tissue remains limited. This gap underscores the need for studies using patient-derived samples to validate findings from in vitro models and to explore the clinical relevance of these epigenetic alterations in tumor progression.

The unique histone modification patterns of TNBC, once fully characterized, may distinguish it from subtypes like luminal A- and HER2-enriched breast cancers, highlighting new possibilities for subtype-specific therapies [9]. Current epigenetic treatments, including DNA methyltransferase (DNMT) and histone deacetylase (HDAC) inhibitors, show clinical promise [10]. Histone methyltransferase (HMT) inhibitors correspond to the third generation of epigenetic drugs capable of writing or deleting epigenetic information [11,12]. The EZH2 inhibitor, targeting H3K27me3, is the first and only HMT inhibitor that was approved by the FDA in 2020 for the treatment of certain cancers [13,14]. Emerging targets like H3K79me1/2/3 (DOT1L inhibitors) and H3K9me2 (G9a/EHMT2 inhibitors) are showing potential in preclinical studies of breast cancer [15,16,17].

In this study, the global expression of histone modifications, histone modifiers, and oncohistone mutations were characterized in BC tissues using immunohistochemical (IHC) staining. Expression patterns were validated across two independent cohorts and compared to those in healthy tissue samples and BC cell lines. Subtype-specific histone modifications were identified, with several significant ones showing strong associations with the TNBC subtype, tumor grade, and stage. Treatment with a small-molecule epigenetic inhibitor significantly impaired cell growth in both positive and negative estrogen receptor cell lines. Transcriptomic and pathway analyses further confirmed distinct responses between the two cell line types. Together, these findings highlight specific histone modifications as critical markers and drivers of advanced or accelerated breast cancer progression and establish histone modifiers as promising therapeutic targets in BC.

## 2. Results

### 2.1. Pathological Scoring Identified Key Histone Biomarkers Across BC Molecular Subtypes

**Figure 1:**
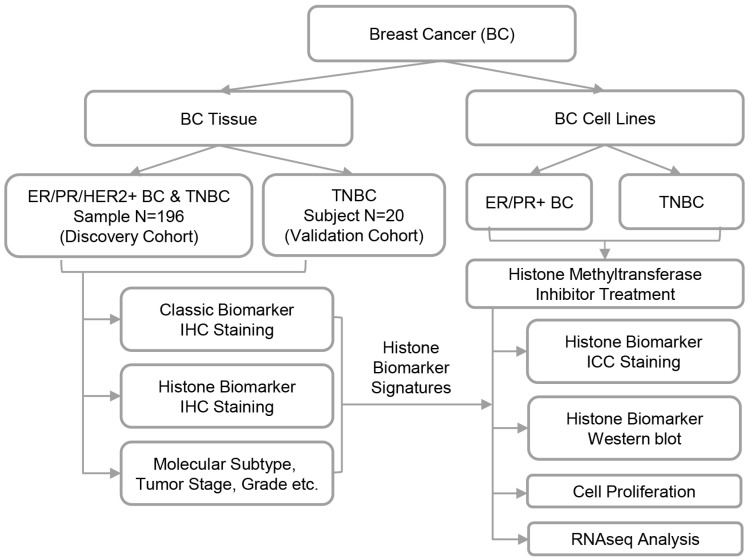
Workflow diagram depicting the identification of histone biomarker signatures in breast cancer.

**Figure 2:**
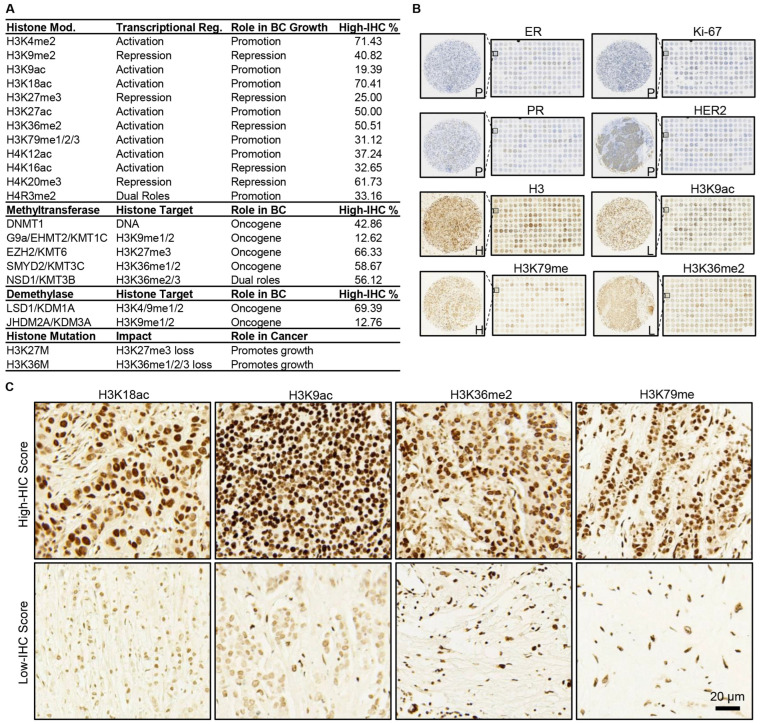
Pathological staining and scoring of BC tissue samples from the discovery cohort. (**A**) The histone biomarkers analyzed in this study are listed. IHC scoring results for the discovery cohort are shown as the percentage of tissue samples with high IHC scores (high IHC%). (**B**) Low-power IHC images display the expression of four classic BC biomarkers (with nuclear counterstain) and four representative histone biomarkers (without nuclear counterstain). (**C**) High-power IHC images illustrate representative histone biomarker expression at both high and low IHC scores. Staining results are annotated as P (positive for classic biomarkers) and H or L (high or low IHC scores for histone biomarkers).

To characterize the global expression of histone biomarkers in tissue samples, IHC staining was initially performed on two cohorts of BC patients (Figure 1). The analysis included 12 histone modifications, 7 histone modifiers, 2 oncohistone mutations, and histone H3 as a control signal (Figure 2A). The histone modifications and modifiers selected in this study are recognized for their roles in driving dysregulated gene expression patterns in breast cancer and will, henceforth, be termed histone biomarkers (Table S1) [9]. The first cohort referred to as the discovery cohort, comprised 196 tissue spots (Figure 2B). These were derived from 98 patients (Table 1), with duplicate spots collected per patient. The mean patient age was 50.4 years. Most patients were classified as grade 2 (80.6%) and clinical tumor stage II (67.3%). In terms of TNM staging, 48.0% were categorized as T2 and 68.4% were identified as N0. Each IHC staining spot of the histone biomarkers was scored by combining the area and density of the dyed region assessed by Image J. The samples were then categorized into two classes: high IHC score and low IHC score (Figure 2C).

**Figure 3:**
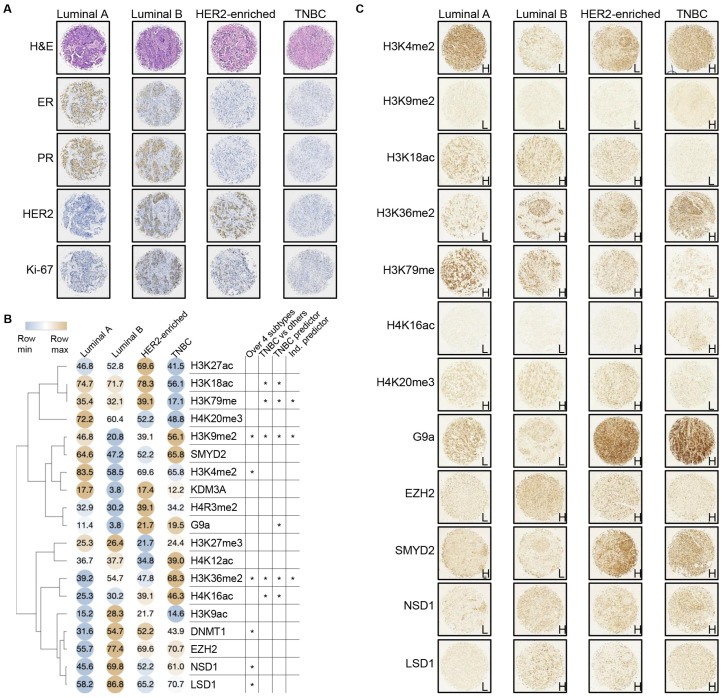
Distribution of histone biomarker levels across BC molecular subtypes. (**A**) Representative H&E and IHC images depicting the four BC molecular subtypes in the discovery cohort, classified based on the results of classic IHC biomarkers, as shown. (**B**) Heatmap and hierarchical clustering of the high IHC% of each histone biomarker across the molecular subtypes. Numbers within the colored circles represent the high IHC% for the corresponding biomarker. Asterisks (*) denote the statistical significance (*p* < 0.05) of four statistical analyses (Table 2 and Table 3): (1) the distribution of histone biomarkers across 4 molecular subgroups; (2) the distribution of histone biomarkers between triple-negative breast cancer (TNBC) vs. other molecular subtypes; (3) the univariate regression analysis performed for each histone biomarker as a predictor of IHC score groups; and (4) multivariate regression for each histone biomarker as an independent predictor. (**C**) Representative IHC images showing the expression of selected histone biomarkers across molecular subtypes. Staining results are annotated as H (high IHC score) and L (low IHC score).

The molecular subtypes of BC tissues (luminal A, luminal B, HER2-enriched, and TNBC) in the discovery cohort were determined by analyzing IHC staining patterns for ER, PR, HER2, and Ki-67 (Figure 3A). The heatmap of the high IHC% suggests that high IHC scores are not uniformly distributed among the molecular subgroups (Figure 3B). In TNBC, H3K9me2, H3K18ac, H3K79me (me1/2/3), and H4K20me3 exhibited a distinct expression pattern compared to the other three molecular subtypes (Figure 3C). Despite sharing consistent high or low IHC scores, there was a noticeable variation in expression levels across these biomarkers, highlighting subtype-specific epigenetic differences. Statistical analyses were conducted to assess the significance of the distribution of histone biomarker levels. Initially, chi-square tests were performed to assess the difference in the distribution of histone biomarkers across sample subgroups defined by clinical characteristics, as described in Table 1. As presented in Table 2 and Figure 3B, six histone biomarkers (H3K9me2, H3K4me2, H3K36me2, DNMT1, NSD1, and LSD1/KDM1A) showed significant differences across four molecular subtypes. H3K9me2, H3K36me2, and H3K79me (me1/2/3) and two acetylation markers (H3K18ac and H4K16ac) exhibited significant differences in TNBC compared to other molecular subtypes.

Logistic regression analysis was then conducted to explore the relationship between IHC scores (high IHC vs. low IHC) and histone biomarkers (Table 3), excluding the oncohistone mutations that were not detectable in any of the tissue samples (Figure S1). Univariate regression was performed to evaluate the association of each histone biomarker with the IHC score individually. Histone biomarkers with *p*-values below 0.05 in the univariate analysis were subsequently included in the multivariate regression model. The analysis highlighted H3K9me2, H3K36me2, and H3K79me (me1/2/3) as independent predictors of altered IHC scores in TNBC compared to other molecular subtypes. G9a, the only histone modifier that exhibited significant differences in TNBC, was determined to be a dependent predictor. Furthermore, H3K9me2 and H3K36me2, along with H3K4me2 and others, were also identified to be independent predictors for the luminal A and luminal B subtypes.

### 2.2. Key Histone Biomarkers Serve as Indicators of Advanced Tumorigenesis in BC

**Figure 4:**
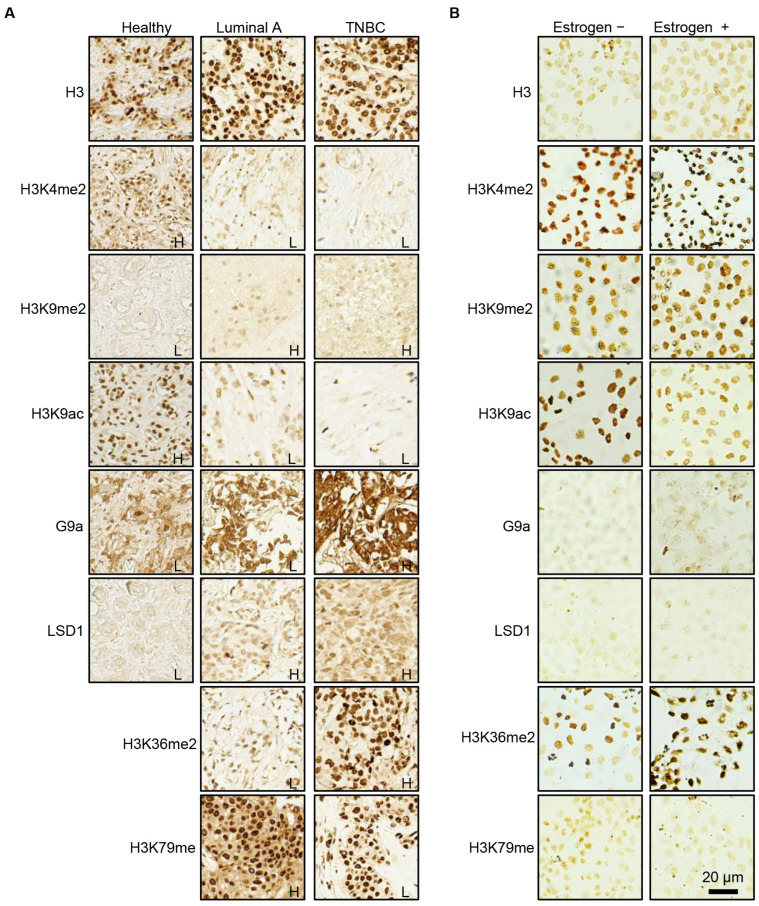
IHC of TNBC-specific histone biomarker signatures in tissue and cell lines. (**A**) High-power IHC image representatives of TNBC-specific histone biomarker signatures in adjacent healthy breast tissue (Healthy), luminal A breast cancer tissue, and TNBC tissue. (**B**) Immunocytochemical (ICC) staining image representatives of TNBC-specific histone biomarker signatures in luminal A cell lines (MCF-7) without or with estrogen treatment. Staining results are annotated as H or L (high or low IHC scores).

Next, we aimed to evaluate the expression levels of selected histone biomarkers in adjacent healthy breast tissue compared to breast cancer tissue. Due to material limitations, the analysis focused on histone H3, three specific histone modifications (H3K4me2, H3K9me2, and H3K9ac), and two corresponding modifiers (G9a and LSD1). Representative IHC images of luminal A breast cancer and TNBC are displayed side by side (Figure 4A). The IHC images of two additional independent TNBC predictors, H3K36me2 and H3K79me, are presented alongside LSD1. Histone biomarkers and corresponding modifiers exhibited clustered expression patterns, as demonstrated in the heatmap analyses (Figure 3B). Interestingly, histone biomarkers commonly associated with transcriptional repression (H3K9me2, G9a, and LSD1) were more highly expressed in TNBC compared to luminal A and were also elevated in breast cancer tissue compared to healthy tissue. In contrast, the expression of biomarkers associated with transcriptional activation (H3K4me2 and H3K9ac) followed a decreasing trend, with the lowest levels observed in TNBC, intermediate levels in luminal A, and the highest levels in healthy tissue.

The MCF-7 cell line treated with estrogen is a well-established luminal A breast cancer model for studying estrogen-driven regulation. A positive transcriptional response to estrogen is central to the biology of ER-positive breast cancer and serves as a hallmark of disease progression and treatment response (Figure S2). To further explore the identified key histone biomarkers, their global expression was assessed via ICC staining in estrogen-treated MCF-7 cells. Notably, the histone biomarkers H3K9me2 and G9a, out of the five markers characterized above in tissue samples, showed increased expression in estrogen-treated MCF-7 cells compared to estrogen-deprived cells (Figure 4B). Additionally, several other methylation and acetylation markers exhibited an altered expression in this cellular model (Figure S3), indicating a complex epigenetic landscape in ER-responsive regulation in breast cancer. These findings reinforce the association of H3K9me2 and G9a with advanced tumorigenesis in breast cancer and underscore their roles in promoting estrogen-dependent tumor growth and progression.

### 2.3. Validation of Altered Global Histone Modification in TNBC Tissue

As the role of G9a and H3K9me2 in ER-positive breast cancer has become better understood, the focus of this study shifted toward further exploring the significance of key histone biomarkers in TNBC. To validate these findings, a second breast cancer cohort was established, comprising 20 tissue slides from 20 TNBC cases. The clinical characteristics of the validation cohort are summarized, with no significant differences observed between the discovery and validation cohorts (Table 1). Each tissue sample was stained for 4 histone biomarkers, and the IHC results were scored and classified into IHC-high and IHC-low categories, as described previously (Table 4). The high IHC percentages of TNBC-independent predictors (H3K9me2, H3K36me2, H3K79me) and modifier G9a in the validation cohort are provided in Table 4, alongside corresponding metrics for TNBC and luminal A cases in the discovery cohort. Significant differences (*p* < 0.05) were observed in the high IHC% of all three independent predictors when comparing at least one TNBC cohort to luminal A, but no significant difference was found between validation TNBC and discovery TNBC. This finding confirmed the agreement of the significance of key histone biomarkers between two TNBC cohorts and further highlighted the distinction between TNBC and luminal A, in addition to the results described above comparing TNBC with all non-TNBC subtypes.

**Figure 5:**
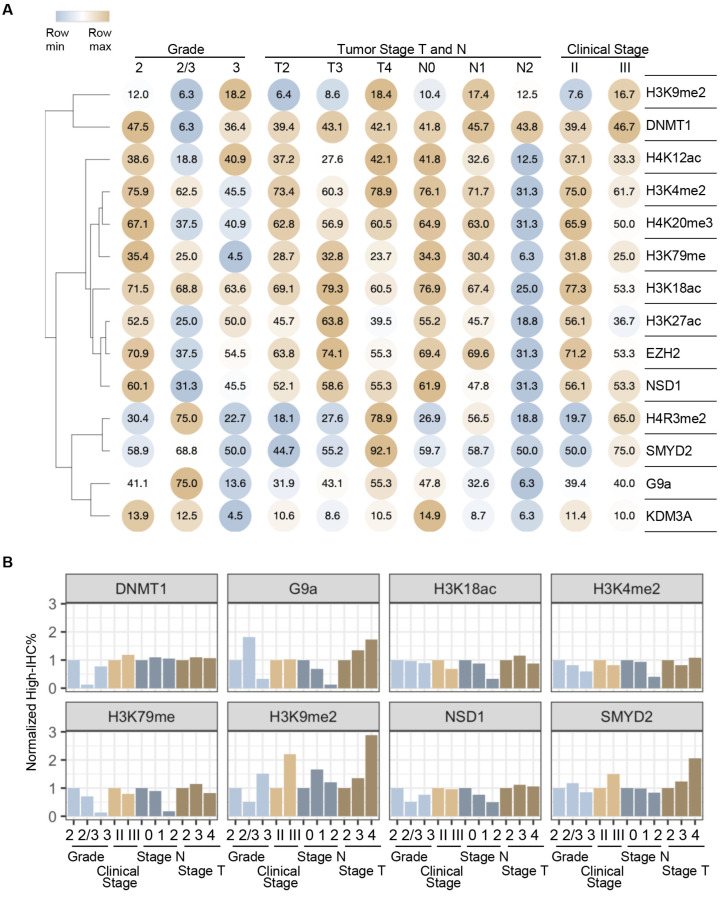
Correlation between histone biomarker levels and tumor grade/stage. (**A**) Heatmap depicting the high IHC% for selected histone biomarkers across tumor grades and stages. Numbers within the colored circles represent the specific high IHC% for each biomarker. Only selected histone biomarkers with significantly different distributions (*p* < 0.05, as shown in Table 2) across at least two grades or stage subgroups are shown. (**B**) Bar plots illustrating the normalized high IHC% of selected histone biomarkers across tumor grades and stages. Normalization was performed using the lowest grade (Grade 2) and stage (N0 or T2) as references. Only histone biomarkers selected in (**A**) that demonstrated significant differences between molecular subtypes are displayed. SMYD2 was included as a control, showing no differential expression across subtype subgroups.

Among the three histone biomarkers validated in this study, H3K9me2 was selected for further analysis. One reason for this selection was that while the roles of G9a and H3K9me2 have been extensively studied in ER-positive breast cancer, their involvement in TNBC remains largely unexplored despite strong prior associations. Another reason is that global H3K9me2 signals were significantly linked to tumor grade, as well as T and N stages (Table 2, Figure 5A). Notably, a gradual increase in the H3K9me2 level (high IHC%) was observed with a higher tumor grade or stage (T4 > T3 > T2) (Figure 5B). This pattern suggests that H3K9me2 may be associated with more aggressive or advanced breast cancer status or subtypes, such as TNBC, which are characterized by higher proliferation rates and poorer clinical outcomes.

### 2.4. G9a Inhibition Disrupts Proliferation and Signaling Pathways in Breast Cancer Cell Lines

**Figure 6:**
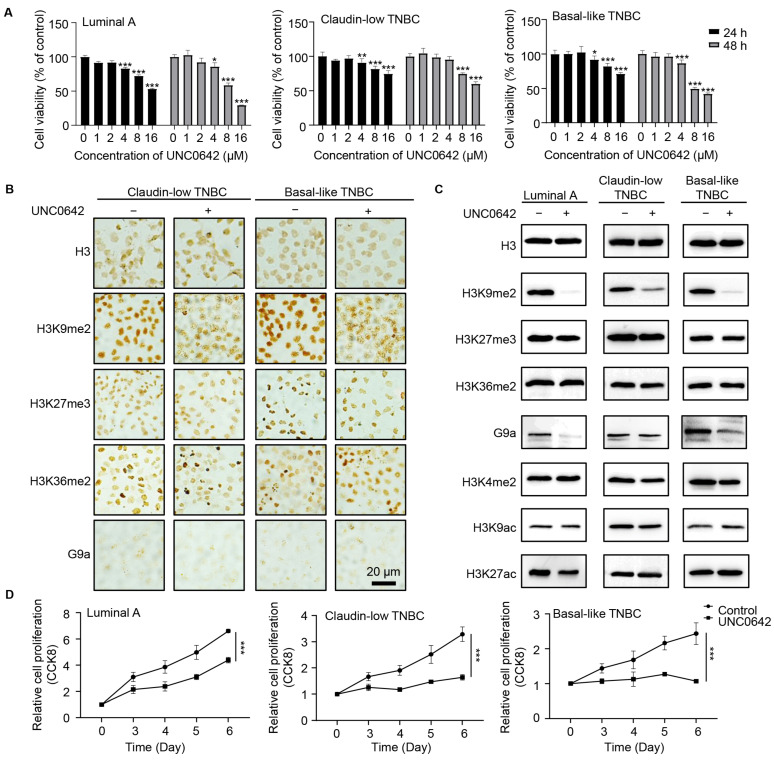
Cell proliferation is impaired in TNBC cell lines following G9a inhibition. (**A**) The cell viability of three breast cancer cell lines (MCF-7 as luminal A, MDA-MB-231 as claudin-low TNBC, and MDA-MB-468 as basal-like TNBC) under treatment using the small-molecule G9a inhibitor UNC0642 was assessed using the Cell Counting Kit-8 (CCK-8) assay to optimize the treatment conditions. (**B**,**C**) ICC staining (**B**) and Western blotting (**C**) were performed to assess the expression of H3K9me2 and other key histone modifications in three cell lines that were treated with UNC0642. (**D**) The cell proliferation of three breast cancer cell lines was assessed using CCK-8 assay. *, *p* < 0.05; **, *p* < 0.01; ***, *p* < 0.001.

UNC0642, a novel small-molecule inhibitor targeting the catalytic activity of G9a, was evaluated in MCF-7 (luminal A), MDA-MB-231 (claudin-low TNBC), and MDA-MB-468 (basal-like TNBC) cell lines. Cell viability assays were performed to optimize the dose and treatment duration (Figure 6A). Finally, non-cytotoxic concentrations of UNC0642 (2 µM) were applied in all subsequent experiments. This inhibitor demonstrated significant efficacy in reducing H3K9me2 levels in all three cell lines, as confirmed by both pathological staining and Western blotting analysis (Figure 6B,C). Other histone modifications, such as H3K4me2, or alternative modifications at the same residue, such as H3K9ac, remained unaffected, indicating the high specificity of UNC0642. Interestingly, G9a protein levels were also reduced in basal-like TNBC cells, which exhibited a more pronounced decrease in H3K9me2 compared to claudin-low TNBC cells. UNC0642 treatment resulted in marked growth inhibition across all tested cell lines, with the most significant effects observed in basal-like TNBC cells, followed by claudin-low TNBC cells, and moderate effects in luminal A cells (Figure 6D).

**Figure 7:**
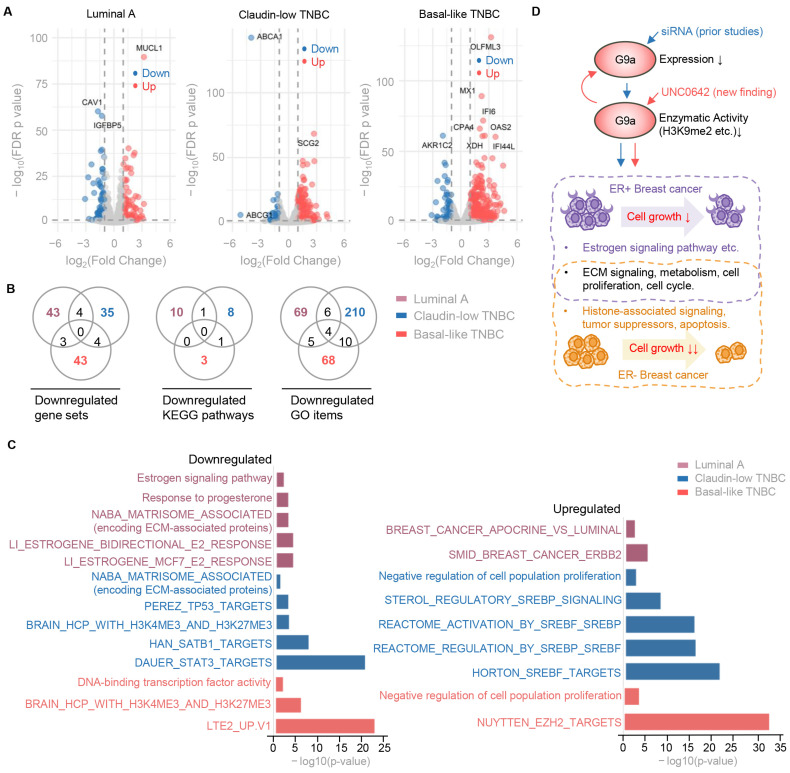
Transcriptomic analysis of BC cell lines treated with G9a inhibition. (**A**) Volcano plots showing differentially expressed genes (DEGs) in each cell line after inhibitor treatment, with up-regulated and down-regulated genes annotated by color. The *x*-axis represents log2 fold change, and the *y*-axis indicates −log10 FDR *p*-value. (**B**) Venn diagrams illustrating the overlap of significantly downregulated gene sets, Kyoto Encyclopedia of Genes (KEGG) pathways, and gene ontology (GO) items among the three cell lines, highlighting shared and cell-line-specific pathways affected by the inhibitor. (**C**) Bar plots of representative enriched KEGG pathways, GO items, and gene sets either down-regulated or up-regulated in BC cell lines as indicated. The *x*-axis represents the −log10 FDR *p*-value. (**D**) A working model illustrates the effects of G9a knockdown and inhibition in breast cancer cell lines, integrating findings from prior studies (blue arrows) and the current work (red arrows). Both siRNA-mediated knockdown and small-molecule inhibitor-mediated inhibition demonstrate how reducing G9a activity impairs cell growth in ER-positive (annotated in purple) and ER-negative (annotated in orange) breast cancer cell lines.

In line with the expected effects of losing the repressive histone marker H3K9me2, the number of up-regulated DEGs was five-fold higher than down-regulated DEGs in two ER-negative cell lines (Figure 7A and Figure S4, Table S2). In contrast, ER-positive MCF-7 cells showed a balanced number of up- and down-regulated DEGs, aligning with prior reports that G9a mediates direct methylation on ERα that is functionally linked to breast cancer progression [17,18] (Figure 7A and Figure S4, Table S2). To further understand the impact of UNC0642, we performed pathway, GO, and GSEA analyses to evaluate gene group and pathway-level responses. The results revealed both shared and distinct transcriptional responses across the three cell lines, highlighting subtype-specific mechanisms of action for the inhibitor (Figure 7B and Figure S4). In MCF-7 cells, estrogen-responsive pathways and gene sets were exclusively down-regulated, supporting previous findings that G9a acts as an ERα coactivator [17] (Figure 7C, Tables S3 and S4). Interestingly, G9a inhibition in MDA-MB-231 cells led to a transcriptional response characterized by the up-regulation of oncogenes such as SREBF and down-regulation of tumor suppressor genes such as TP53, STAT3, and SATB1 (Figure 7C, Tables S3 and S4). Cell proliferation pathways and extracellular matrix (ECM)-associated gene sets are consistently down-regulated in both ER-negative and ER-positive cells, emphasizing the shared growth-suppressive effects of G9a inhibition and underscoring its significant therapeutic potential across these breast cancer subtypes (Figure 7C).

## 3. Method

### 3.1. Study Design

To identify and evaluate the potential of 21 pathological biomarkers in breast cancer, this study used tissue microarray technology, allowing for the high-throughput molecular analysis of 196 tissue samples from a discovery cohort of 98 breast cancer patients (Figure 1). The IHC profiling results of TNBC were then validated by 20 additional tissue blocks from a validation cohort of 20 TNBC patients. Eligible participants of both cohorts were women aged 35–70 years with a first primary diagnosis of breast cancer (Table 1). The distributions of clinical characteristics and traditional pathological biomarker results are shown for both the discovery and validation cohorts, as provided by the commercial vendors of BC tissues. Based on IHC assessments of ER, PR, HER2, and Ki-67, breast cancer samples in this study were classified into 79 luminal A cases, 53 luminal B cases, 23 HER2-enriched cases, and 41 TNBC cases in the discovery cohort, as well as 20 TNBC cases in the validation cohort. Histone biomarker signatures identified from tissue samples were subsequently evaluated in BC cell lines. To explore their functional relevance, the selected HMT inhibitor, the G9a inhibitor UNC0642, was applied to assess its effects on cell proliferation, impacted gene expression, and signaling pathways. All study procedures were approved by the Institutional Ethics Committee and the Institutional Review Board of Northwest University (approval number: 200402001).

Histone biomarkers strongly associated with molecular types of breast cancer (BC) were identified through immunohistochemical (IHC) staining in tissue and cell line samples. The functional roles of histone biomarker signatures were further investigated using cell line models treated with small molecule inhibitors targeting histone methyltransferases.

### 3.2. Tissue Specimens

We collected 216 tissue samples from commercial sources for this study. A high-density breast cancer tissue microarray (chip number F1961101 B06-F1961101 B25) was purchased from Xi’an Bioaitech Co., Ltd. (Xi’an, China). Each chip included two 1 mm tissue cores from 98 patients with various molecular subtypes, forming a discovery cohort of 196 samples. A validation cohort was established using 20 TNBC tissue slides procured from Shanghai Xinchao Co., Ltd. (Shanghai, China). (with the sample codes listed in Table S1). Clinical characteristics and biomarker data were available for all tissue samples (Table 1).

### 3.3. Cell Culture

Three breast cancer cell lines—MCF-7 (luminal A), MDA-MB-231 (claudin-low TNBC), and MDA-MB-468 (basal-like TNBC)—and two T-lymphocytic leukemia cell lines (HPB-ALL and LOUCY) were used in this study. The origin and catalog number of cell lines are listed in Table S1. Three BC cell lines were cultured in Dulbecco’s modified Eagle’s medium (DMEM). HPB-ALL and LOUCY cells were cultured in RPMI-1640. In both media, 10% fetal bovine serum and 1% penicillin–streptomycin were added. The above cells were grown in a humidified 5% CO_2_ incubator at 37 °C. For estrogen treatment, cells were starved in phenol red-free DMEM supplemented with 10% charcoal-stripped FBS for 2 days, and 73.4 mM of estrogen was added to the medium (3 and 6 h for gene expression studies and 6 h for immunocytochemical (ICC) experiments) before the cells were collected. The detailed list of reagents and kits used in this method can be found in Table S1.

### 3.4. IHC and ICC

IHC analysis was performed using the streptavidin–biotin–peroxidase complex method to evaluate 20 histone biomarkers (including H3) of interest. Tissue chips and slides were processed by deparaffinization, antigen retrieval, blocking with goat serum, and incubation with primary antibodies overnight at 4 °C. Secondary antibodies and DAB were used for detection, followed by counterstaining and mounting. Anti-H3 served as a control. Details of antibodies, reagents, and kits are provided in Table S1. ICC was performed using the same principles as IHC. Cells were fixed in 4% paraformaldehyde, permeabilized with 0.25% Triton X-100, and blocked with 10% goat serum. Primary antibodies were incubated overnight, followed by the incubation of secondary antibodies and DAB detection.

### 3.5. Cell Viability and Proliferation Assay

Cell viability was assessed using the Cell Counting Kit-8 (CCK-8). Cells were seeded in 96-well plates (5 × 10^3^/well), treated with UNC0642 or DMSO (Control), and incubated. After treatment, the 10 µL CCK-8 solution was added, and OD450 was measured. For proliferation assays, cells were treated with 2 µM UNC0642 or DMSO and analyzed over six days using the same method. A comprehensive list of reagents and kits, along with their references where applicable, is provided in Table S1.

### 3.6. Western Blotting

Proteins from BC cells, which were treated with 2 µM UNC0642 or DMSO for 48 h, were extracted using the RIPA buffer, quantified using the BCA assay, and separated by SDS-PAGE. After transferring to PVDF membranes, blots were incubated with primary and secondary antibodies. Signals were detected using enhanced chemiluminescence, with H3 as a loading control. The details of antibodies, reagents, and kits are listed in Table S1.

### 3.7. RNA Isolation and Sequencing

Total RNA from BC cells collected after 4 days of treatment with 2 µM UNC0642 or DMSO was extracted using Trizol and assessed for purity and integrity. RNAseq libraries were prepared using the TruSeq RNA Sample Prep Kit and sequenced on an Illumina NovaSeq 6000 platform. Clean reads were analyzed using STAR aligner and DESeq2 to identify differentially expressed genes (DEGs). Genes in breast cancer cell lines that displayed at least a two-fold difference in gene expression between comparison groups (fold change > 2 or <−2, FDR *p* < 0.05) were considered significant DEGs and carried forward in the analysis. Pathway and gene ontology (GO) enrichment analysis was performed via an integrated platform KOBAS 3.0 and gene set enrichment analysis (GSEA) using MSigDB gene sets within selected collections of H, C2, C4, and C6. Bioinformatics analysis was performed and visualized using R version 3.6.1 or Python version 3.7.9. The detailed list of reagents and kits used in the method can be found in Table S1.

### 3.8. Quantitative Real-Time PCR (qRT-PCR)

Quantitative real-time PCR was performed using Bio-Rad real-time PCR systems. Total RNA (1 µg) was reverse-transcribed into cDNA using the Hifair III 1st Strand cDNA Synthesis SuperMix for qPCR. mRNA expression levels were quantified using Hieff^®^ qPCR SYBR Green Master Mix, with relative expression calculated by the 2^−ΔΔCt^ method. Each sample was tested in triplicate, and primers, reagents, and kits are listed in Table S1.

### 3.9. IHC Evaluation and Cutoff Determination

Histone expression was quantified using integrated optical density (IOD) values from Image J (Media Cybernetics Inc., Rockville, MD, USA) analysis [19]. Receiver operating characteristic (ROC) curve analysis was used to determine cutoff scores for each histone, distinguishing high and low expressions [20]. Clinical features, including age, grade, T stage, N stage, tumor stage, and receptor status, were classified for analysis. For each histone biomarker, the points of maximum sensitivity and specificity were selected based on the area under the curve. Samples were classified as having low expressions if their integrated optical density was below the cutoff and a high expression if they were equal to or above the cutoff. Clinical features used for stratification in ROC analysis included the following: age (≥50 vs. <50 years), tumor grade (Grade 2 vs. 2–3/3), T stage (T1/T2 vs. T3/T4), N stage (N0 vs. N1/N2), tumor stage (Stage I vs. Stage II/III), receptor status (ER/PR/HER2 negative vs. positive), and Ki-67 Index (<14% vs. >14%). These cutoff values provided a basis for categorizing histone expression and analyzing its association with breast cancer subtypes and clinical outcomes.

### 3.10. Statistical Analysis

Statistical analyses were conducted using SPSS (version 25.0, SPSS Inc., Chicago, IL, USA) and GraphPad Prism 9 (San Diego, CA, USA). *t*-tests, chi-square tests, and logistic regression were used to analyze data.

## 4. Discussion

Significant efforts have been dedicated to systematically profiling histone modifications and the enzymes that regulate them in ER-positive BC [21]. These studies have been pivotal in driving the development and clinical application of the first two generations of epigenetic drugs, including DNMT inhibitors and HDAC inhibitors, with a third generation targeting HMT now emerging. However, similar insights into histone modification landscapes and their therapeutic potential remain scarce for TNBC, a subtype known for its aggressive and invasive nature. A greater challenge in developing epigenetic drugs is their specificity, which requires emphasis on identifying biomarkers capable of predicting how individual patients respond to these therapies. This study aimed to address this critical knowledge gap by unveiling key histone biomarker patterns in BC with a specific focus on TNBC. Among the >20 histone biomarkers evaluated, our findings revealed significant alterations in four specific histone modification/modifier pairs (H3K4me2/LSD1, H3K9me2/G9a, H3K36me2/NSD1, DNMT1) across the four major BC molecular subtypes. These findings align with the established evidence supporting epigenetic therapy in driving ER-positive breast cancer progression [11,13]. For example, LSD1 was proposed as a therapeutic target, not only as a standalone approach but also in combination with hormonal therapies [22]. Given the availability of LSD1 inhibitors in preclinical and clinical stages, our results further support their exploration as a promising additional therapy in breast cancer [14,23].

In our analysis with TNBC cohorts, three histone methylation signals (H3K9me2, H3K36me2, and H3K79me) emerged (and were validated) as being independently associated with TNBC, underscoring their potential role as subtype-specific epigenetic markers and therapeutic targets. This result aligns with published reports emphasizing the roles of methylations on H3K36 and H3K79 in TNBC epigenetic regulation. For instance, distinct patterns of H3K36me3 have been observed in TNBC cell lines, particularly in relation to androgen receptor pathway activity [7]. Additionally, the chromatin state marked by H3K4me3 and H3K79me2 at loci such as AFAP1-AS1 has been identified as a hallmark of TNBC, particularly in driving active transcription programs in these cells [7]. Independent studies have confirmed that both NSD3, a histone methyltransferase responsible for H3K36me2, and DOT1L, which methylates H3K79, are highly expressed in the MDA-MB-231 TNBC cell line [24,25]. The inhibition or knockdown of these histone writers leads to a reduction in H3K36me2 and H3K79me levels, respectively. This reduction in histone modifications is associated with a significant decrease in cell proliferation, migration, and invasion. Notably, in the context of TNBC, these effects are often linked to the reversal of epithelial-to-mesenchymal transitions (EMTs), which is a key process that contributes to increased metastatic potential and the aggressive nature of cancer cells [26]. Here, our findings provide direct evidence from multiple cohorts of human tissue to support observations previously made in cell line studies and underscore the therapeutic potential of targeting histone writers like NSD3 and DOT1L in TNBC treatment strategies.

Mutations in histone H3 (such as H3K36M and H3K27M), so-called “oncohistones”, have been identified as significant contributors to tumorigenesis in certain cancers and, thus, were investigated in this study [27]. H3K36M is known to drive skeletal tumors like chondroblastoma by dominantly inhibiting H3K36 methylation, leading to transcriptional dysregulation and altered differentiation [28]. The H3K36M oncohistone primarily inhibits several H3K36-specific methyltransferases, leading to a reduction in all methylation states of H3K36 [29]. Similarly, the H3K27M mutation disrupts the repressive H3K27me3 mark, resulting in widespread epigenetic reprogramming and aberrant cellular proliferation or tumorigenesis [30]. In our study, neither H3K36M nor H3K27M mutations were detected in any of the BC tissue samples examined by histological staining. These findings suggest that while H3 mutations play pivotal roles in certain malignancies, their contribution to BC pathogenesis, particularly in the context of our cohort, appears minimal. This highlights the need for further exploration into the unique histone modification patterns that define BC subtypes.

The most substantial finding presented here is that H3K9me2 deposited by G9a serves as an independent predictor, with its histological staining results effectively distinguishing TNBC from other breast cancer subtypes. Furthermore, the inhibition of G9a drastically impairs the proliferation of breast cancer cell lines from various subtypes, including luminal A, claudin-low TNBC, and basal-like TNBC, underscoring its potential as a therapeutic target across these subtypes. Compared to H3K9me3, which is often linked to the formation of heterochromatin defining stable and highly repressive chromatin states, H3K9me2 is less stable and associated with dynamic repression [7]. Its writer G9a has broader substrate specificity, targeting both histone and non-histone proteins, such as p53, CDYL, and ERα [31]. G9a is well-recognized as a coactivator in ER-positive breast cancer, where its inhibition reactivates p53 and induces necroptosis, highlighting its dual roles in tumor progression [32]. Mechanistically, G9a directly methylates ERα, thereby enhancing its transcriptional activity on genes that drive cell growth and survival [17]. The experimental depletion of G9a in breast cancer cells and colorectal cancer stem cells has been shown to suppress motility and disrupt ECM organization, underscoring its broader role in cancer cell dynamics [33,34]. Importantly, to our knowledge, this study represents the first transcriptomic analysis of TNBC cells following G9a inhibition by a small-molecule inhibitor (Table S5). The findings reveal pathways that partially overlap with but are distinct from those identified in prior siRNA-mediated G9a knockdown studies [35]. Our transcriptomic analysis of G9a-inhibited BC cells supports previous findings by revealing G9a’s critical role in maintaining ER activity in ER-positive BC cells and promoting ECM signaling pathways in both ER-positive and -negative BC cells (Table S5). While ER signaling is specific to ER-positive cells, EMT pathway regulation appears consistent across both ER-positive and ER-negative breast cancer cells, highlighting G9a’s broader influence on cellular processes irrespective of the estrogen receptor’s status [36,37]. Together, our working model (Figure 7D) illustrates the shared and distinct effects of G9a inhibition and knockdown on breast cancer cell lines. Both approaches impair cell proliferation, with ECM regulation and proliferation pathways commonly affected across all cell lines. However, the impact on tumor-suppressor pathways and apoptosis emerges as a more prominent outcome in ER-negative BC cells. This model highlights the overlapping and context-specific roles of G9a in breast cancer, emphasizing how different intervention strategies can lead to distinct cellular responses.

Given G9a’s involvement in estrogen receptor coactivation, it is considered a potential therapeutic target for ER-positive breast cancer. However, its epigenetic role in ER-negative breast cancer remains uncertain, given the absence of ER signaling in these cancers. Notably, the IHC score of H3K9me2 in our cohorts is significantly higher in higher-grade and later-stage tumors compared to healthy tissue, lower-grade, earlier-stage, or less aggressive subtypes like luminal A. This observation suggests that H3K9me2 may play a more crucial role in the progression of more aggressive cancers or breast cancer that does not have hormone receptors. We also observed in Figure 6C that the G9a inhibitor not only limits G9a’s enzymatic activity but also reduces its expression at the transcriptional level. This dual effect may stem from feedback regulatory mechanisms, where inhibiting enzymatic activity disrupts downstream signaling pathways, ultimately altering gene expression patterns. Consistent with this, our findings show that G9a inhibition has a more profound inhibitory effect on cancer growth in basal-like TNBC > claudin-low TNBC > luminal A BC, highlighting G9a’s broader influence across different BC subtypes via distinct pathways and mechanisms. Additionally, our results reinforce earlier conclusions that G9a inhibitors induce apoptosis in breast cancer cell lines, with a more pronounced tumor volume reduction in MDA-MB-231 cells compared to MCF-7 cells [37]. Although cell proliferation was impaired, our transcriptomic profiling revealed that G9a inhibition in TNBC cell lines uniquely induces the upregulation of oncogene target genes and the downregulation of genes targeted by tumor suppressors such as SATB1 and TP53 [38,39]. This contrasts with prior studies, which reported that G9a inhibitors effectively suppress tumor growth in preclinical models, though our findings diverge in terms of the reactivation of silenced tumor suppressor genes [36,40,41]. This observation suggests that the heightened sensitivity of ER-negative breast cancer cells to G9a inhibitors may be influenced by the epigenetic changes induced by treatment rather than reflecting a direct mechanistic basis.

This study, while providing valuable insights, has several limitations that warrant consideration. The relatively small cohort size and lack of follow-up or survival data impede a comprehensive understanding of the prognostic value of histone biomarkers. Additionally, the scoring methodology, reliant on manual assessment, requires significant labor and experienced technicians and carries the risk of manual error, emphasizing the need for automation through machine learning-driven imaging technology. Furthermore, the absence of in vivo validation limits the ability to confirm the therapeutic potential of G9a inhibitors, highlighting the need for preclinical studies and further investigation into its dualistic functions in breast cancer subtypes.

## 5. Conclusions

The immunohistochemical profiling and transcriptomic analyses presented in this study identified effective subtype-specific epigenetic targets in various BC subtypes, with a particular focus on TNBC. The findings highlight G9a’s multifaceted role in regulating both hormone-driven pathways and tumor suppressor mechanisms, demonstrating its context-dependent influence. This research sheds light on the epigenetic complexity underlying TNBC and provides valuable insights for developing targeted therapeutic strategies, offering a promising avenue to improve outcomes for patients with this challenging breast cancer subtype.

## Figures and Tables

**Table 1:** Characteristics of cohort patients and classic BC biomarker features.

Characteristic Features		Discovery Cohort: All BC Cases (Specimen N = 196)	Discovery Cohort: TNBC Cases (Specimen N = 41)	Validation Cohort: TNBC Cases (Specimen N = 20)	*p* Value *
Mean age (year)		50.4 ± 11.0	49.2 ± 12.2	57.5 ± 12.5	0.017
Grade (%)	1–2	0 (0%)	0 (0%)	1 (5.0%)	0.654
	2	158 (80.6%)	23 (56.1%)	9 (45.0%)	
	2–3	16 (8.2%)	10 (24.4%)	6 (30.0%)	
	3	22 (11.2%)	8 (19.5%)	4 (20.0%)	
T stage (%)	T1	6 (3.0%)	0 (0%)	2 (10.0%)	0.052
	T2	94 (48.0%)	20 (48.8%)	13 (65.0%)	
	T3	58 (29.6%)	8 (19.5%)	3 (15.0%)	
	T4	38 (19.4%)	13 (31.7%)	2 (10.0%)	
N stage (%)	N0	67 (68.4%)	32 (78.0%)	17 (85.0%)	0.546
	N1	46 (23.4%)	5 (12.2%)	0 (0%)	
	N1–2	0 (0%)	0 (0%)	3 (15.0%)	
	N2	16 (8.2%)	4 (9.8%)	0 (0%)	
Tumor stage (%)	I	4 (2.0%)	0 (0%)	2 (10.0%)	0.083
	II	132 (67.3%)	26 (63.4%)	15 (75.0%)	
	III	60 (30.6%)	15 (36.6%)	3 (15.0%)	
ER (%)	Negative	78 (39.8%)	41 (100%)	20 (100%)	NA
	Positive	118 (60.2%)	0 (0%)	0 (0%)	
PR (%)	Negative	96 (49.0%)	41 (100%)	20 (100%)	NA
	Low (<20%)	18 (9.2%)	0 (0%)	0 (0%)	
	High (≥20%)	82 (41.8%)			
HER2 (%)	Negative	158 (80.6%)	41 (100%)	20 (100%)	NA
	Positive	38 (19.4%)	0 (0%)	0 (0%)	
Ki-67 (%)	Low (<14%)	106 (54.1%)	17 (41.5%)	1 (5.0%)	0.003
	High (≥14%)	90 (45.9%)	24 (58.5%)	19 (95.0%)	

* A *t*-test was used to compare the mean age values, while a chi-square test was applied to compare the distribution of TNBC cases across different characteristic groups between the discovery cohort (N = 41) and the validation cohort (N = 20).

**Table 2:** Comparison of histone biomarker levels across sample subgroups defined by patient characteristics.

	Statistical Significance of the Distributions (*p* Value *)							
	Grade	T Stage	N Stage	Tumor Stage	PR	Ki-67	Molecular Subtypes ^#^	TNBC ^##^
H3K4me2	0.009	0.068	0.001	0.073	0.619	0.000	0.014	0.374
H3K9me2	0.001	0.043	0.003	0.052	0.597	0.002	0.003	0.025
H3K9ac	0.368	0.321	0.094	0.359	0.192	0.002	0.235	0.387
H3K18ac	0.741	0.251	0.000	0.001	0.003	0.843	0.142	0.024
H3K27me3	0.422	0.939	0.046	0.421	0.099	0.620	0.978	0.919
H3K27ac	0.111	0.079	0.018	0.045	0.045	0.566	0.158	0.219
H3K36me2	0.077	0.431	0.094	0.406	0.473	0.006	0.022	0.010
H3K79me	0.012	0.002	0.072	0.007	0.001	0.159	0.160	0.029
H4K12ac	0.273	0.005	0.055	0.028	0.046	0.462	0.988	0.791
H4K16ac	0.545	0.328	0.787	0.367	0.020	0.906	0.111	0.036
H4K20me3	0.007	0.225	0.032	0.031	0.015	0.101	0.058	0.055
H4R3me2	0.001	0.000	0.001	0.000	0.96	0.386	0.896	0.880
DNMT1	0.005	0.037	0.898	0.042	0.592	0.003	0.047	0.879
G9a	0.001	0.185	0.000	0.421	0.989	0.649	0.057	0.110
EZH2	0.012	0.073	0.008	0.019	0.267	0.012	0.062	0.502
SMYD2	0.508	0.000	0.758	0.001	0.627	0.010	0.152	0.294
NSD1	0.048	0.141	0.028	0.190	0.160	0.001	0.043	0.481
LSD1	0.107	0.245	0.082	0.182	0.418	0.001	0.006	0.834
KDM3A	0.466	0.000	0.395	0.000	0.165	0.287	0.109	0.904

* Chi-square test comparing the distribution of high IHC% across sample subgroups defined by each characteristic provided in Table 1. Only selected characteristics that show statistically significant differences (*p* < 0.05) of at least one histone biomarker in the grouping of samples are presented here. ^#^ Comparison across four sample subgroups: Lumina A, Lumina B, HER2-enriched, and TNBC subtypes. ^##^ Comparison between TNBC and non-TNBC subgroups.

**Table 3:** Logistic regression analysis for histone biomarkers associated with individual molecular subtypes.

	Univariate Regression (*p* Value)				Multivariate Regression (*p* Value)			
	LuminalA	LuminalB	HER2-Enriched	TNBC	LuminalA	LuminalB	HER2-Enriched	TNBC
H3K4me2	0.003	0.016	0.833	0.375	0.002	0.160		
H3K9me2	0.160	0.001	0.861	0.027		0.039		0.012
H3K9ac	0.224	0.058	0.762	0.389				
H3K18ac	0.282	0.810	0.383	0.026				0.057
H3K27me3	0.933	0.781	0.701	0.919				
H3K27ac	0.530	0.630	0.052	0.221				
H3K36me2	0.01	0.474	0.784	0.012	0.016			0.013
H3K79me	0.284	0.861	0.380	0.033				0.001
H4K12ac	0.899	0.931	0.795	0.791				
H4K16ac	0.073	0.654	0.482	0.038				0.121
H4K20me3	0.014	0.812	0.318	0.057	0.000			
H4R3me2	0.951	0.590	0.519	0.880				
DNMT1	0.01	0.043	0.339	0.879	0.170	0.056		
G9a	0.519	0.281	0.367	0.013				0.191
EZH2	0.01	0.049	0.727	0.503	0.172	0.769		
SMYD2	0.170	0.048	0.502	0.295		0.017		
NSD1	0.015	0.02	0.685	0.482	0.394	0.409		
LSD1	0.006	0.002	0.645	0.834	0.186	0.015		
KDM3A	0.091	0.036	0.481	0.904		0.077		

**Table 4:** TNBC-specific histone biomarker signatures across subtypes and cohorts.

	H3K9me2	H3K36me2	H3K79me	G9a
#1: Discovery Luminal A (N = 79)	37 (46.8%)	31 (39.2%)	28 (35.4%)	9 (11.4%)
#2: Discovery TNBC (N = 41)	23 (56.1%)	28 (68.3%)	7 (17.1%)	8 (19.5%)
*p* value (#2 vs. #1) *	0.010	0.003	0.036	0.226
#3: Validation Cohort (N = 20)	15 (75.0%)	12 (60.0%)	6 (30.0%)	3 (15.0%)
*p* value (#3 vs. #1) *	0.024	0.094	0.647	0.659
*p* value (#3 vs. #2) *	0.153	0.522	0.247	0.667

* Chi-square test provided to compare the distribution of the high IHC% across sample subgroups as indicated.

## Data Availability

Raw and processed files of RNA sequences (FASTQ format) supporting the findings of this study were deposited in the National Center for Biotechnology Information (NCBI) Gene Expression Omnibus (GEO) under accession number GSE283819.

## Supplementary Materials

The following supporting information can be downloaded at https://www.mdpi.com/article/10.3390/ijms26020770/s1.
