# Supplementary material for: Immunohistochemical Profiling of Histone Modification Biomarkers Identifies Subtype-Specific Epigenetic Signatures and Potential Drug Targets in Breast Cancer
Source: Int J Mol Sci. 2025 Jan 17;26(2):770. doi: 10.3390/ijms26020770 (PMC11765579; doi:10.3390/ijms26020770)
Supplement: Supplementary file 1 [file ijms-26-00770-s001.zip › Supplementary Legend.pdf]

Supplementary Figure S1. Two oncohistone mutations were assessed using immunohistochemical (IHC) staining in breast cancer (BC) tissues. The LOUCY cell line was used as a positive control for H3K36M and a negative control for H3K27M, while HPB-ALL served as a positive control for H3K27M and a negative control for H3K36M.

Supplementary Figure S2. Relative mRNA levels of ERα-responsive genes TFF1, PR, and GREB1 were measured by quantitative real-time PCR (qRT-PCR) in MCF-7 cells treated with estrogen for 3 and 6 h. The mRNA level was normalized to GAPDH and relative levels were calculated using 0 h as reference. Error bars indicate the mean ± SEM. of three replicates. Significant fold changes are indicated as follows: *, $p < 0.05$; **, $p < 0.01$; ***, $p < 0.001$ (Student's t-test).

Supplementary Figure S3. Immunocytochemical (ICC) staining images of additional histone biomarkers in MCF-7 cell lines without or with estrogen treatment.

Supplementary Figure S4. Venn diagrams illustrating the overlap of significantly regulated differently expressed gene (DEG), gene sets, Kyoto Encyclopedia of Genes (KEGG) pathways and Gene Ontology (GO) items among the three cell lines.

Table S1 List of reagents and supplies used in this study

Table S1a List of tissue sample codes used in this study

Table S1b List of cell lines used in this study

Table S1c List of reagents and kits used in this study

Table S1d List of antibodies used in this study

Table S1e List of PCR primers used in this study

Table S1f Background of histone biomarkers and the references

Table S2 DEG lists identified from breast cancer cell lines treated with G9a inhibition

Table S2a List of DEGs identified in MCF-7 cells, n=193

Table S2b List of DEGs identified in MDA-MB-231 cells, n=214

Table S2c List of DEGs identified in MDA-MB-468 cells, n=473

Table S3 Gene sets identified from breast cancer cell lines treated with G9a inhibition

Table S3a Regulated gene sets identified in MCF-7 cells ($p < 0.05$), n=100

Table S3b Regulated gene sets identified in MDA-MB-231 cells ($p < 0.05$), n=93

Table S3c Regulated gene sets identified in MDA-MB-468 cells ($p < 0.05$), n=100

Table S4 Pathways identified from breast cancer cell lines treated with G9a inhibition

Table S4a Regulated KEGG pathways identified in MCF-7 cells ($p < 0.05$), n=19

Table S4b Regulated KEGG pathways identified in MDA-MB-231 cells ($p < 0.05$), n=38

Table S4c Regulated KEGG pathways identified in MDA-MB-468 cells ($p < 0.05$), n=47

Table S4d Regulated GO items identified in MCF-7 cells ($p < 0.05$), n=156

Table S4e Regulated GO items identified in MDA-MB-231 cells ($p < 0.05$), n=353

Table S4f Regulated GO items identified in MDA-MB-468 cells ($p < 0.05$), n=418

Table S5 Comparison between the present study and previously reported studies on G9a downregulation in breast cancer cell lines
