# Supplementary figures and images for: Immunohistochemical Profiling of Histone Modification Biomarkers Identifies Subtype-Specific Epigenetic Signatures and Potential Drug Targets in Breast Cancer
Source: Int J Mol Sci. 2025 Jan 17;26(2):770. doi: 10.3390/ijms26020770 (PMC11765579; doi:10.3390/ijms26020770)

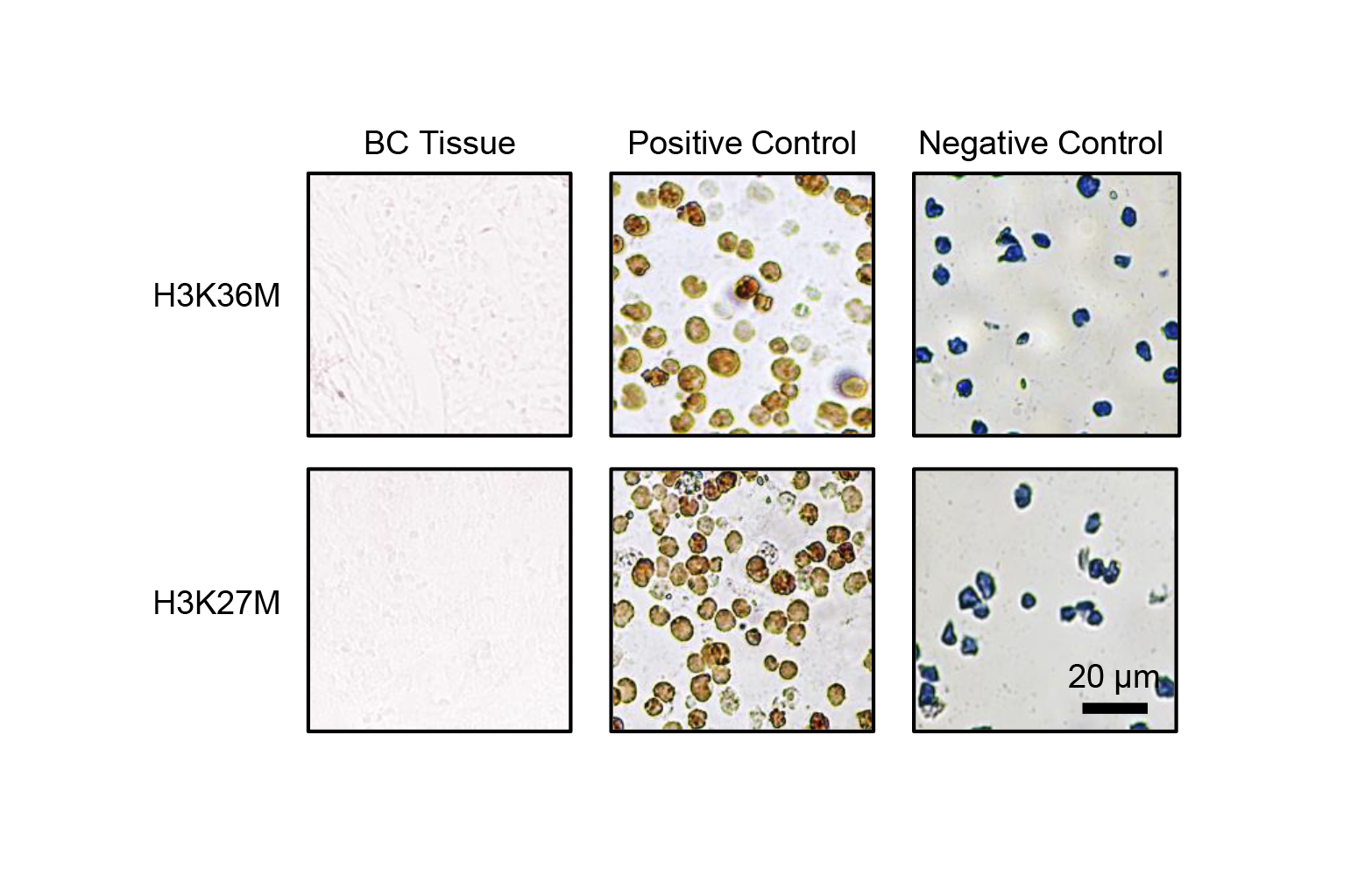

Supplement: Supplementary file 1 [file ijms-26-00770-s001.zip › Figure S1.tif]

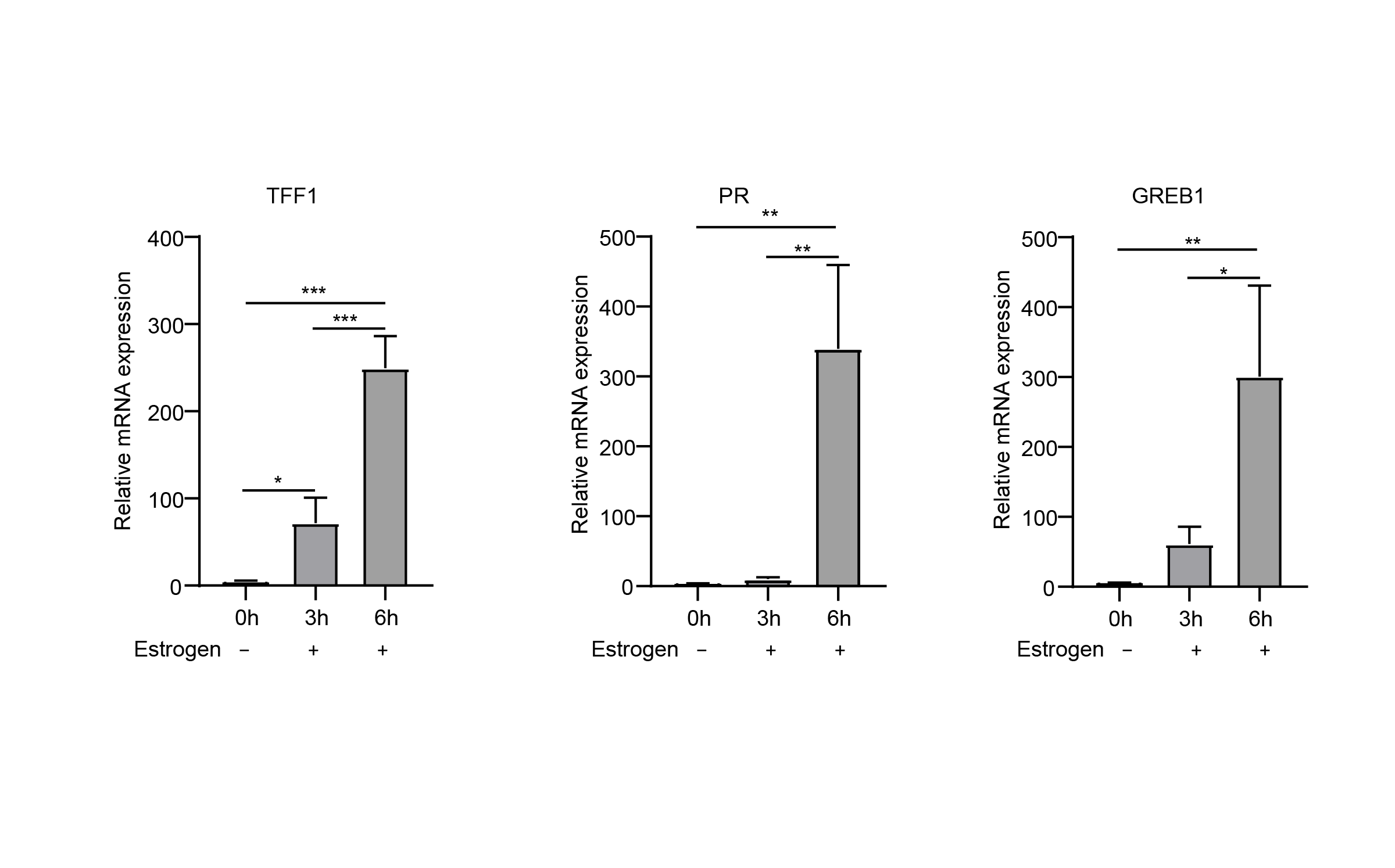

Supplement: Supplementary file 1 [file ijms-26-00770-s001.zip › Figure S2.tif]

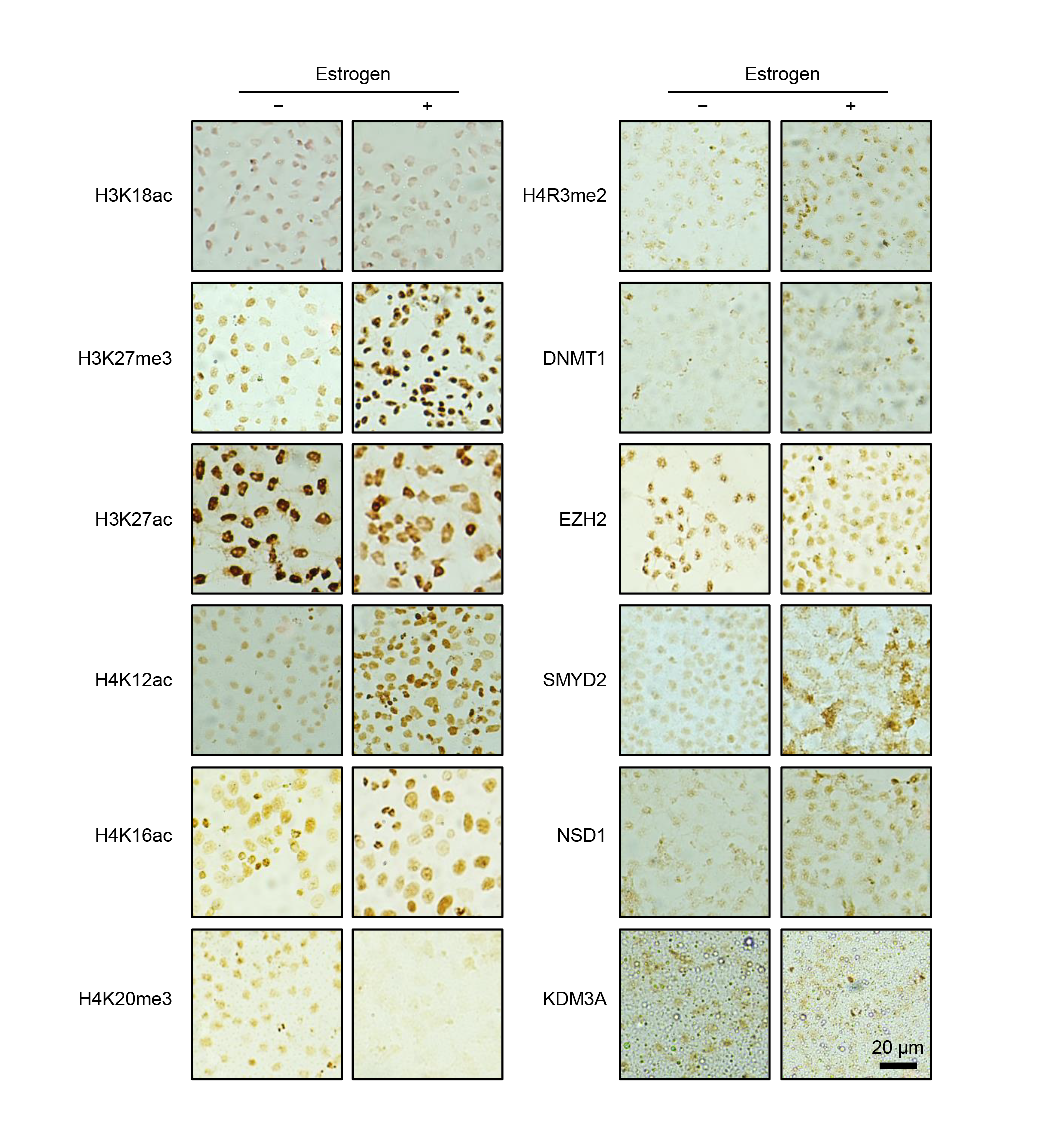

Supplement: Supplementary file 1 [file ijms-26-00770-s001.zip › Figure S3.tif]

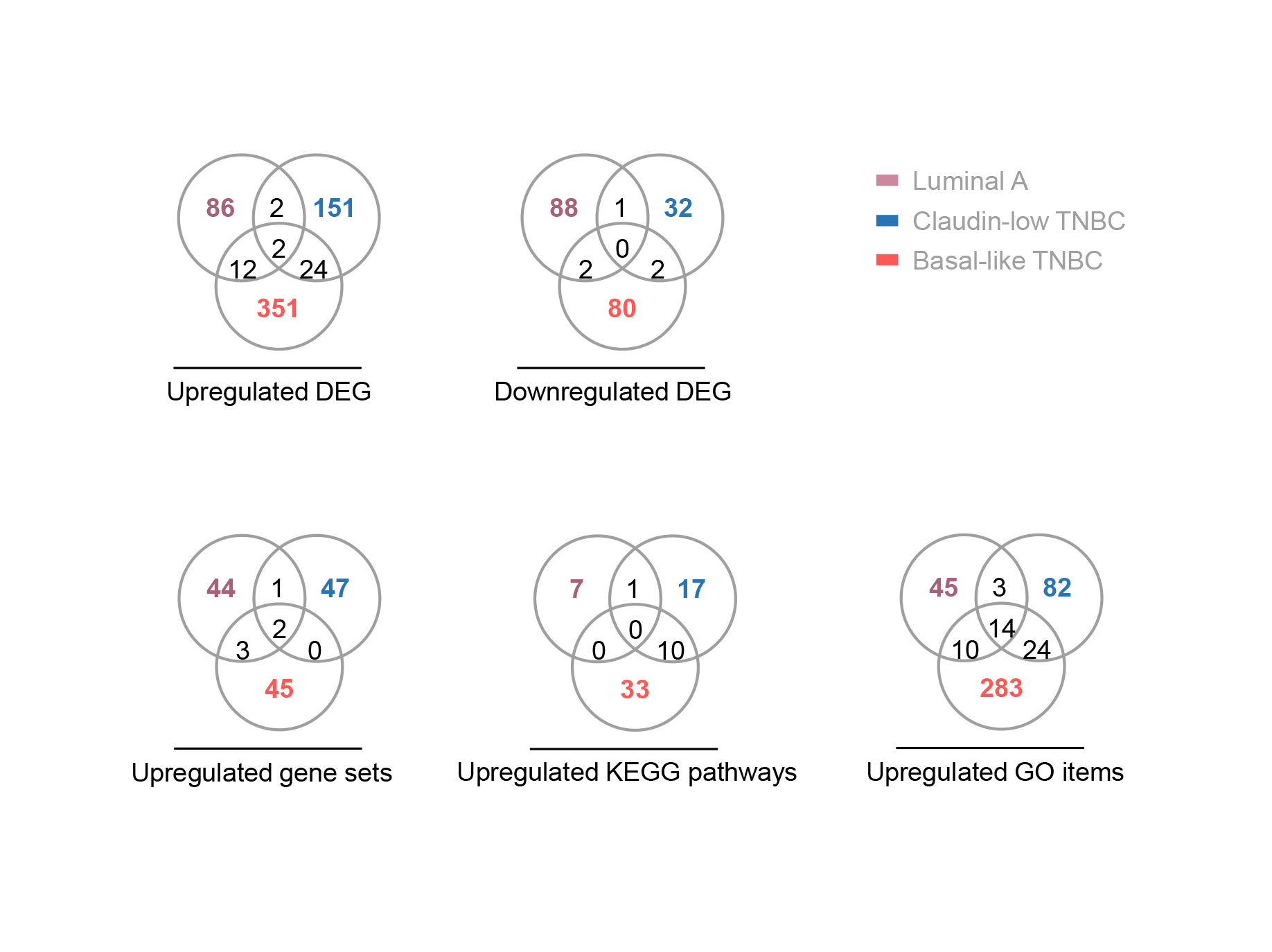

Supplement: Supplementary file 1 [file ijms-26-00770-s001.zip › Figure S4.tif]
